# Our Journal for 1881

**Published:** 1881-01-20

**Authors:** 


### *OUR JOURNAL FOR* 1881.

As our volume dates with the January issue, this is No. 1 of Vol. 11. Thus it will be seen that we are entering upon our eleventh year. In the last ten years the RECORD has held its own, passing through those trying initiatory struggles in which so many noble crafts have sunk—manfully encountering, and successfully passing through the ordeal of the panic of 1873 and the hard years immediately succeeding, until now it is able to state to its readers that all is well, and the outlook for the future more flattering than for many years.

During the present year renewed efforts will be made to improve the already popular features of the Journal in all its departments. Arrangements have been made to enhance the interest of the Original department, a number of able gentlemen having engaged to write for us. The article in the present issue by Prof. Powell, our senior editor, will, it is believed, prove interesting, especially to young practitioners. Upon closely analyzing the "Conversations" it will be seen that the Professor has sought to inculcate a thought or principle, either moral, ethical or medical, in every paragraph. He expects to continue the conversations, embodying practical thoughts and suggestions upon various topics. His long experience as a writer, a teacher, and a practitioner, well fits him for the task which he has assumed. W.

---

*The Principles and Methods of Therapeutics.*—By Alphonse Gubler, M. D., Professor of Therapeutics in the Faculty of Medicine of Paris, etc. Translated from the French. One volume 8vo. In press. Ready March 1st, 1881.

Gubler may be said to have been the most distinguished exponent of scientific therapeutics—in the best sense of the term—of this generation. Following Trousseau in the professional chair, and a pupil of that great teacher, he took a long step in advance of his master, and may be said to have developed the only method of therapeutics which reconciles the empirical and clinical art of medicine with the demands of exact and logical science. His labors created a new epoch in professional practice in France, and in all other countries where they have become known have made a profound impression on the professional mind.

